# Comparison of haemoglobin assessments by HemoCue and two automated haematology analysers in young Laotian children

**DOI:** 10.1136/jclinpath-2017-204786

**Published:** 2017-12-02

**Authors:** Guy-Marino Hinnouho, Maxwell A Barffour, K Ryan Wessells, Kenneth H Brown, Sengchanh Kounnavong, Bigphone Chanhthavong, Kethmany Ratsavong, Chidchamai Kewcharoenwong, Sonja Y Hess

**Affiliations:** 1 Department of Nutrition, Program in International and Community Nutrition, University of California, Davis, California, USA; 2 Nutrition and Global Development, Bill & Melinda Gates Foundation, Seattle, Washington, USA; 3 National Institute of Public Health, Vientiane, Laos; 4 Faculty of Associated Medical Sciences, Centre for Research and Development of Medical Diagnostic Laboratories, Khon Kaen University, Khon Kaen, Thailand

**Keywords:** paediatric haematology, diagnosis, epidemiology, evaluating instrument

## Abstract

**Background:**

Haemoglobin (Hb) assessment by Hemocue is used widely for anaemia screening in both adults and children. However, few studies have compared the diagnostic accuracy of Hemocue with an automated haematology analyser in young children.

**Aim:**

To compare Hb concentrations by Hemocue Hb301 and two automated haematology analysers in young children in rural communities of Lao PDR.

**Methods:**

Capillary blood was collected from 6-month-old to 23-month-old children (n=1487) for determination of Hb concentration by Hemocue Hb301. On the same day, venous blood was collected for complete blood count using one of two haematology analysers (XT-1800i, Sysmex, and BC-3000Plus, Mindray Medical International). In a subsample of children (n=129), venous Hb was also measured by HemoCue Hb301. Agreement between the two methods was estimated using Bland-Altman plots.

**Results:**

Mean capillary Hb by Hemocue was significantly higher than mean venous Hb by haematology analysers combined (108.4±10.3 g/L vs 102.3±13.1 g/L; P<0.001), resulting in a significantly lower anaemia prevalence (Hb <110 g/L) by Hemocue (53.7% vs 73.9%; P<0.001). The Bland-Altman assessment of agreement showed a bias of 6.1 g/L and limits of agreement were −11.5 g/L to 23.7 g/L. Mean venous Hb concentration by Hemocue Hb301 (113.6±14.0 g/L) was significantly higher than mean capillary Hb concentration by Hemocue Hb301 (110.0±10.7; P=0.03 g/L), which in turn was significantly higher than mean venous Hb concentration by the Mindray BC-3000Plus (102.3±17.4 g/L).

**Conclusion:**

Capillary and venous Hb concentrations assessed by Hemocue Hb301 showed poor agreement compared with venous Hb by automated haematology analysers, resulting in significantly different anaemia prevalences.

## Introduction

Anaemia is a major public health concern, affecting 43% of young children and 38% of pregnant women globally.^1^ Primary causes of anaemia include micronutrient deficiencies, parasitic infections, such as malaria and hookworm, and inherited haemoglobin (Hb) disorders.^2–5^Anaemia has several negative consequences on health and may cause low birth weight, preterm birth and perinatal, neonatal and maternal mortality during pregnancy.^6^ In addition, iron deficiency anaemia has been linked with poor psychomotor development of children and reduced physical performance in adults.^7^

Hb concentration is the most commonly used indicator of anaemia at the individual and population level^8^ and is used for both screening and evaluating the impact of intervention programmes.^9^ Numerous methods are available for measuring Hb concentration,^10–14^ of which the cyanmethemoglobin method is considered the reference method by the International Committee for Standardization in Hematology^10^ and is recommended by the World Health Organization (WHO).^15^ However, this analytical method is fairly time consuming and therefore relatively costly.^16^ In clinical laboratory settings, Hb concentration is generally assessed by automated haematology analysers,^12 13^ which are very reliable and accurate, but expensive and not transportable to the field. In field settings where resources are limited, the HemoCue device has been used extensively because it is portable, easy to use and relatively inexpensive. Moreover, the HemoCue device requires only a small drop of capillary or venous blood and provides an immediate numerical Hb value.^17^

Multiple studies have examined the accuracy and precision of HemoCue results compared with automated haematology analysers in different adult populations, and findings from these studies are mixed.^18–25^ Several factors may explain the discrepancy between studies, including measurement errors, blood sampling site (capillary vs venous blood),^26^ analytical setting (laboratory vs field) and population characteristics (healthy adults, pregnant women, blood donors, ill patients, etc).

To date, few published data exist on the accuracy of HemoCue versus an automated haematology analyser in children both in laboratory^27–29^ and field settings.^25 30^ To our knowledge, only one study included very young children.^28^ This study, implemented in 11-month-old to 36-month-old children in immunisation clinics in east London, found that Hb concentrations measured by HemoCue were consistently greater than Hb levels measured by a haematology analyser (Coulter counter). Because the anaemia prevalence of children under 5 years of age is assessed by HemoCue in most national surveys and considering the reported inconsistency of the relative accuracy and reliability of this method,^7 17^ the present study aimed to: (1) compare Hb concentrations measured by HemoCue Hb301 and automated haematology analysers in young children in rural communities of Lao PDR, (2) compare the estimated anaemia prevalence derived from these two methods, (3) determine the accuracy of HemoCue Hb301 using both venous and capillary blood samples compared with that of an automated haematology analyser using venous blood and (4) compare the Hb measurements provided by the three different project-owned HemoCue Hb301 devices.

## Methods

### Study population

Data were drawn from the Lao Zinc Study, a community-based randomised controlled trial implemented in rural communities of Khammouane Province in Lao PDR (registered at www.ClinicalTrials.gov; NCT02428647). The study protocol and the consent procedure were approved by the National Ethics Committee for Health Research (Lao PDR), Institutional Review Board of the University of California, Davis (USA), and the Khon Kaen University Ethics Committee in Health Research (Thailand). Prior to any examination, written informed consent (documented by either a signature or a fingerprint in the presence of a neutral witness) was obtained from one of the child’s primary caregivers (mother, father or legal guardian). In a first set of analyses of the present paper, we used data from a convenience subsample of 1487 children, 6–23 months of age at baseline whose Hb concentrations were assessed during study enrolment (September 2015 through August 2016; objectives 1 and 2). All data available were used for the present methodological comparison, which was considered adequate based on sample size recommendations for the Bland-Altman method by Lu *et al*.^31^ Retrospective sample size calculation showed that our sample size of n=1487 allowed us to estimate the bias with a 95% CI of 0.91 g/L and the Bland-Altman limits of agreement with a 95% CI of 1.6 g/L. For logistical reasons, the venous blood samples were analysed by two different haematology analysers, as described in more detail below. In a second set of analyses involving 129 children, 15–32 months of age, we compared venous and capillary Hb concentrations measured during the endline assessment (January through March 2017; objective 3). In a third set of analyses involving 29 children, 15–32 months of age, we compared the Hb measurements by each of the three project-owned HemoCue Hb301 devices (November 2016; objective 4).

### Estimation of Hb concentration

Non-fasting capillary and venous blood samples were collected within a few minutes of each other by trained phlebotomists, with the child in a seated position. Blood was drawn from the left side of the body whenever possible, and the sampling sites were cleaned with alcohol and left to air dry for a few seconds.

For capillary blood collection, the tip of the left middle finger was pricked with a lancet (Haemolancet Plus, HTL-STREFA, Poland), and the first drop of blood was wiped away with cotton. The fingertip of the left middle finger was used whenever possible because previous studies have reported a large within-subject variation in Hb concentration from the left to the right hand.^9^ A standard Hb301 microcuvette was used to collect the second drop of blood, and the cuvette was then wiped on the sides to remove any excess blood and immediately inserted into the HemoCue Hb301 device (HemoCue AB, Angelholm, Sweden) for analysis. The HemoCue Hb301 system determines the Hb concentration by measuring the absorbance of whole blood at an Hb/HbO2 isosbestic point. The device uses a double wavelength measuring method, 506 nm and 880 nm, for compensation of turbidity.^32^ The results are displayed numerically in g/dL at the time of the measurement. In the present study, Hb results were recorded on an electronic device and manually in a notebook.

Venous blood was drawn from an antecubital, dorsal metacarpal or great saphenous vein into an EDTA tube (S-Monovette (K3 EDTA), Sarstedt AG & Co, Nümbrecht, Germany) from children who were not acutely ill with fever or reported diarrhoea. Right after collection, the tube was gently inverted 8–10 times and placed into a cold box at 4°C–8°C. The sample was transported the same day either to Nakhon Phanom Hospital, Thailand, or to the project field laboratory in Nongbok, Lao PDR. A complete blood count (CBC) was performed to estimate Hb concentrations using one of two automated haematology analysers: XT-1800i by Sysmex (n=633; at the Nakhon Phanom Hospital, Thailand) or BC-3000Plus by Mindray Medical International Ltd (n=854; at the project field laboratory, Nongbok, Lao PDR) for different aspects of the Lao Zinc Study. Using a unique diode laser bench, the Sysmex XT-1800i fluorescent flow cytometry uses a cyanide-free, sodium lauryl sulfate method and provides the sensitivity needed for measuring and differentiating cell types in whole blood and body fluid samples. Using a cyanide-free reagent, the BC-3000Plus uses a colorimetric method to determine Hb concentrations. Hb measurements with the HemoCue Hb301 and the two automated haematology analysers were performed in accordance with the manufacturers’ operating manual.

For objective 3, venous Hb was measured using the HemoCue Hb301 device in a subsample of children to explore the differences between venous and capillary Hb concentrations by the HemoCue Hb301 device. After gently inverting the EDTA tube 8–10 times, approximately 50 µl of whole blood was pipetted and placed on a microscopic slide. The blood drop was subsequently aspirated into a standard Hb301 microcuvette and immediately inserted into the HemoCue Hb301 device to determine the venous Hb concentration.

To compare the Hb measurements provided by the different project-owned HemoCue Hb301 devices (objective 4), venous Hb was measured in a subsample of children (n=29), using three different devices. For each measurement, approximately 50 µl of whole venous blood collected in an EDTA tube was placed on a microscopic slide and aspirated into a standard Hb301 microcuvette. A new blood drop was placed on a new microscopic slide for each HemoCue device.

Of the three different HemoCue Hb301 devices, the HemoCue Hb301 #1 and #2 were used in the field for routine capillary Hb concentration determinations for the main trial (objectives 1 and 2). HemoCue Hb301 #1 was used in the substudy of the 129 children (objective 3). HemoCue Hb301 #3 was a back-up device, kept in the laboratory and was only used for the HemoCue comparison substudy (objective 4).

### Quality control

Quality control checks were performed weekly for the HemoCue Hb301 devices (quality control checks for HemoCue Hb301 #3 were performed weekly only when the substudy was implemented), weekly for the project-owned BC-3000Plus haematology analyser and daily for the XT-1800i haematology analyser by Sysmex at the Nakhon Phanom Hospital using the CBC-3D haematology control and a commercial control sample from Meditop Company, respectively. For external quality control, the hospital-based haematology analyser was also qualified by the External Quality Assessment Schemes in Clinical Microscopy from the Faculty of Medical Technology, Mahidol University, and by the Bureau of Laboratory Quality Standards, Ministry of Public Health, Thailand.

The Eurotrol Hb301 Control (levels 1 and 2) was used to verify the precision and accuracy of the HemoCue Hb301 devices. The expected Hb values provided by the manufacturer were 71.0±8.0 and 130.0±12.0 g/L for levels 1 and 2, respectively.

### Statistical analysis

Data are presented as mean±SD or percentage when appropriate. Results of the automated haematology analysers were analysed combined and individually, as described in more detail below. Paired t-tests and McNemar tests were used to compare the difference between mean Hb concentrations and the anaemia prevalences, respectively, between the different methods of assessment. Analysis of variance followed by Bonferroni corrected post hoc pairwise tests was used to compare the average bias between age groups. The correlation between the HemoCue Hb301 and the automated haematology analysers was plotted graphically to assess a possible linear association between the Hb values estimated by the two methods,^33^ and the Pearson’s correlation coefficient (r_p_) was calculated.

The prevalence of anaemia was determined using the Hb cut-off for children 6–59 months of age (Hb <110 g/L).^8^ The automated haematology analysers were considered the reference method against which the sensitivity and specificity of the HemoCue Hb301 were determined.

The concordance correlation coefficient (ρc) or Lin’s coefficient,^34^ which combines measures of both precision and accuracy, was used to determine whether the observed data significantly deviated from the line of perfect concordance (ie, the line at 45 degrees). The bias and limits of agreement between the HemoCue Hb301 and the automated haematology analysers were estimated using Bland-Altman plots.^35^

Several complementary analyses were undertaken to assess the robustness of our findings. First, we repeated the same analyses with each automated analyser individually to assess the accuracy of the HemoCue Hb301 device against each automated analyser. It is important to note that, for this analysis, each comparison was done in a different subgroup of children. Second, we repeated the main analyses by age group (6–11, 12–17 and 18–24 months) to explore whether the accuracy of the HemoCue Hb301 device differed by age group. Third, in a subsample of 129 children, we measured both venous Hb and capillary Hb using the HemoCue Hb301 and venous Hb using the automated haematology analyser at the field site (Mindray BC-3000Plus). Finally, we compared the Hb measurements provided by the different project owned HemoCue Hb301 devices using venous blood of 29 children.

Analyses were undertaken using Stata 14 (StataCorp 2015, College Station, Texas, USA). Reported P values are two-tailed, and P values <0.05 were considered to be statistically significant.

## Results

### Capillary Hb concentration by HemoCue Hb301 versus venous Hb concentration by two automated analysers combined

A total of 1487 children were included in this set of analyses. Their mean age±SD was 14.7±5.1 months, and 48.7% were female. Of these children, 35.2% (n=523) were 6–11 months, 33.8% (n=502) were 12–17 months and 31.1% (n=462) were 18–24 months.

The mean capillary Hb value obtained by HemoCue Hb301 was significantly greater than the mean venous Hb value obtained by the two automated haematology analysers combined (108.4±10.3 g/L vs 102.3±13.1 g/L; P<0.001; table 1). Accordingly, the prevalence of anaemia in the study population was significantly lower using the HemoCue Hb301 device than the automated haematology analysers combined (53.7% vs 73.9%; P<0.001).

**Table 1:** Haemoglobin concentration and anaemia prevalence in young Laotian children (n=1487) using two different methods of assessment

	Hemocue Hb301	Automated haematology analysers combined*	P value
Haemoglobin concentration, g/L			
Mean±SD	108.4±10.3	102.3±13.1	<0.0001
Range	70–141	34–178	
SE	0.3	0.3	
Anaemia prevalence, n (%)	799 (53.7)	1099 (73.9)	<0.0001
Sensitivity of Hemocue Hb301 (%)	68.7	Ref	
Specificity of Hemocue Hb301 (%)	85.8	Ref	
Agreement of the two methods (%)		71.9	

*XT-1800i by Sysmex and BC-3000Plus by Mindray Medical International.

**Figure 1:**
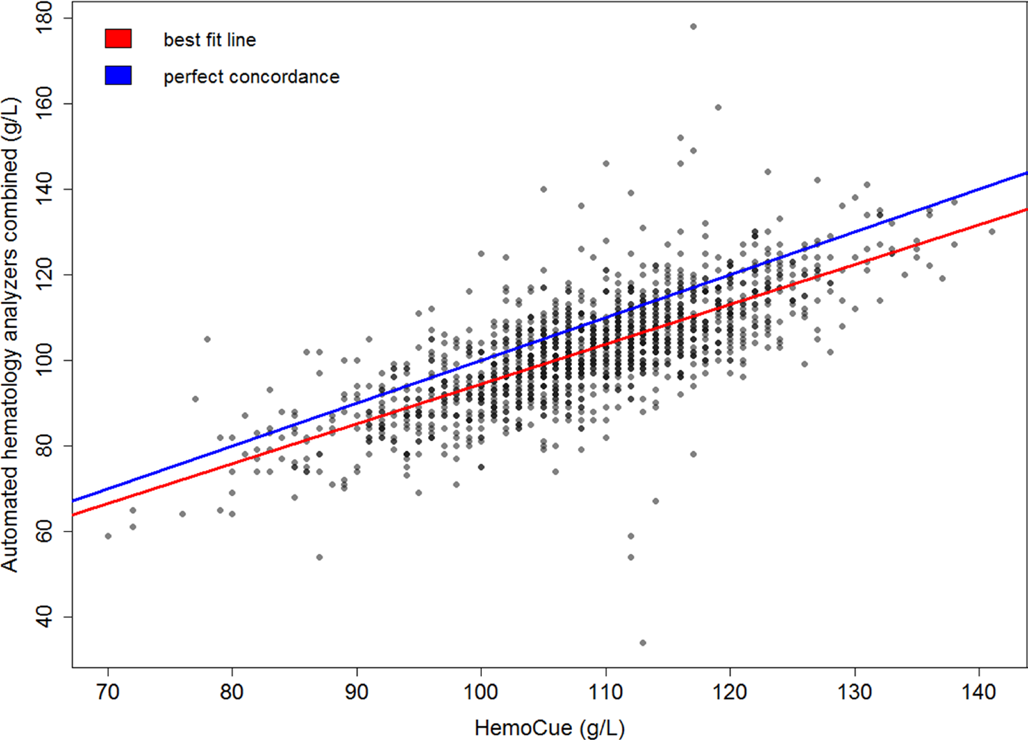
Correlation of Hb concentrations assessed by Hemocue Hb301 and automated analysers combined (XT-1800i by Sysmex and BC-3000Plus by Mindray Medical International Ltd.) and concordance plots between the 2 methods.

**Figure 2:**
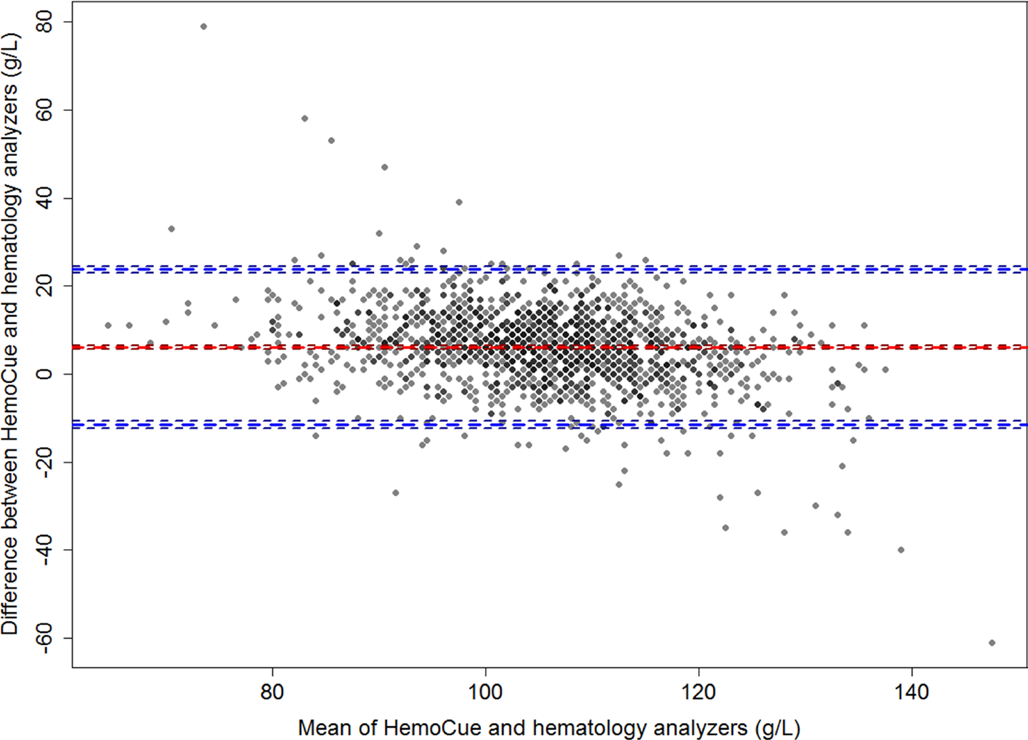
Bland-Altman plot showing agreement in haemoglobin concentration of young Laotian children assessed by HemoCue Hb301 and two automated haematology analysers combined (XT-1800i by Sysmex and BC-3000Plus by Mindray Medical International).

The sensitivity and specificity of the HemoCue Hb301 compared with the two automated haematology analysers combined were 68.7% and 85.8%, respectively, suggesting that the two methods agreed better in non-anaemic children (table 1). The two methods agreed in 71.9% of cases. A linear regression showed a strong correlation between the two methods (correlation coefficient r_p_=0.73; P<0.001). The concordance correlation coefficient was ρc=0.63 (P<0.001) and the reduced major axis and the line of perfect concordance were not aligned (figure 1). Compared with venous Hb concentrations measured by the two automated haematology analysers, the HemoCue Hb301 device appears to overestimate capillary Hb concentrations at lower Hb levels. The Bland-Altman plot revealed an overall bias of 6.1 g/L and the limits of agreement (from −11.5 to 23.7 g/L) indicated that the two methods did not have good agreement (figure 2).

Results of analyses with the automated haematology analysers considered individually showed similar trends (online supplementary table S1 and figures S1 and S2). Mean capillary Hb by Hemocue Hb301 was significantly higher than mean Hb obtained by both the Sysmex XT-1800 (110.7±9.7 g/L vs107.4±12.5 g/L; P<0.001) and the Mindray BC-3000Plus (106.7±10.4 g/L vs 98.4±12.2 g/L; P<0.001). Sensitivity, specificity and agreement of Hemocue Hb301 were slightly better with the Sysmex XT-1800i haematology analyser.

10.1136/jclinpath-2017-204786.supp1 Supplementary data

The mean capillary Hb obtained by HemoCue Hb301 was significantly greater than the mean venous Hb obtained by the two automated haematology analysers combined in all three age groups (all P<0.0001), but the difference between methods was similar in 12-month to 17-month and 18-month to 24-month children (5.9 g/L vs5.0 g/L; P=0.33), but significantly lower than that of children 6–11 months of age (5.9 g/L vs 7.3 g/L; P=0.036 and 5.0 g/L vs 7.3 g/L: P<0.003, respectively) (online supplementary table S2). Regardless of the age group, the box plots comparing the distribution of Hb levels by age group using the two methods (online supplementary figure S3) show a narrower range with HemoCue Hb301 compared with those with automated haematology analysers combined.

### Hb concentration in capillary and venous blood by HemoCue Hb301 versus venous blood by Mindray BC-3000Plus

Mean venous Hb concentration by Hemocue Hb301 was significantly higher than mean capillary Hb concentration by Hemocue Hb301, which in turn was significantly higher than mean venous Hb concentration by the Mindray BC-3000Plus (113.6±14.0, 111.7±10.7 and 102.3±17.4 g/L, respectively; all P<0.05, n=129; table 2). However, it has to be noted that the difference between mean venous and capillary Hb concentration by Hemocue Hb301 was small but clinically significant. The prevalence of anaemia was 36.4%, 41.9% and 65.9%, respectively, in venous blood by Hemocue Hb301, capillary blood by Hemocue Hb301 and venous blood by automated analyser.

**Table 2:** Paired comparisons of haemoglobin concentrations by HemoCue Hb301 (capillary and venous) and by Mindray BC-3000Plus in young Laotian children (n=129)

Comparisons	Hb (mean±SD), g/L	P*	Bias (limits of agreement)	Anaemia prevalence n (%)	P†
Capillary HemoCue	111.0±10.7			54 (41.9)	
versus		0.03	−2.5 (–4.8 to –0.2)		0.21
Venous HemoCue	113.6±14.0			47 (36.4)	
Capillary HemoCue	111.0±10.7			54 (41.9)	
versus		<0.0001	8.7 (6.0 to 11.4)		<0.0001
Venous automated analyser	102.3±17.4			85 (65.9)	
Venous HemoCue	113.6±14.0			47 (36.4)	
versus		<0.0001	11.2 (8.2 to 14.2)		<0.0001
Venous automated analyser	102.3±17.4			85 (65.9)	

*P value for difference of mean Hb concentration.

†P value for anaemia prevalence.

Hb, haemoglobin.

### Hb measurements provided by the three different HemoCue Hb301 devices

In the analyses comparing the results of the three different HemoCue Hb301 devices for 29 venous blood samples (online supplementary table S3), mean Hb did not differ significantly between HemoCue Hb301 #1 and #2 used in the field (112.9±8.8 g/L vs 113.2±6.9 g/L; P=0.96). However, mean Hb concentration obtained with HemoCue Hb 301 #3 (117.1±9.4 g/L) was significantly higher than the mean Hb obtained with both HemoCue Hb301 #1 and #2. There was a good correlation among the three different HemoCue Hb301 devices (r ranged from 0.62 to 0.89). However, based on the Bland-Altman plots, the agreement was good only between the HemoCue Hb301 #1 and #2 with a bias (95% limits of agreement) of −0.28 (−8.23 to 7.68) g/L. We found a within-instrument variation (coefficient of variation (CV)) of 7.7%, 6.1% and 8.0%, respectively, for HemoCue #1, #2 and #3.

### Quality control results

Results from the quality control tests using Eurotrol HB301 control solutions showed that the average concentration was 74.0±3.0 g/L (CV=3.8%) and 131.0±3.0 g/L (CV=2.5%) for levels 1 and 2, respectively, which was within the acceptable range suggested by the manufacturer.

## Discussion

In this study of almost 1500 young children, mean capillary Hb concentrations by HemoCue Hb301 were significantly higher compared with mean venous Hb concentrations determined by two automated haematology analysers (Sysmex XT-1800i and Mindray BC-3000Plus), resulting in a significantly lower anaemia prevalence by Hemocue (53.7% vs 73.9%). Possible reasons for this difference are true physiological differences in Hb concentrations of venous versus capillary blood, differences in accuracy of the analytical instruments and differences in specimen collection and processing prior to analyses. To investigate whether this difference was due to the blood sampling site or the analytical instrument used, we also assessed capillary and venous Hb by HemoCue Hb301 and compared these with venous Hb concentration by the Mindray BC-3000Plus in a substudy of 129 children and found that venous Hb levels were slightly higher than capillary Hb concentrations by HemoCue Hb301 and that both were substantially higher than venous Hb concentrations obtained with the Mindray BC-3000Plus automated haematology analyser, resulting in a significantly lower prevalence of anaemia when the HemoCue Hb301 device was used. Thus, regardless of the type of blood sample, HemoCue Hb301 showed a bias towards higher Hb concentrations compared with an automated haematology analyser.

The instruments used in our study performed well in quality control testing throughout the project. Moreover, the two HemoCue Hb301 devices (#1 and #2) used in the main analyses yielded similar results and the third device (#3) with significantly higher Hb concentrations was not used in any of the comparisons other than the device comparison for the present report. Thus, we trust that our findings derive from accurate and precise instruments. However, it has to be noted that the quality control solutions for Hemocue devises provided by Eurotrol (Burlington, Massachusetts, USA) consider a very wide range acceptable, thus not allowing to properly assess accuracy and precision of Hemocue devices. A reduction in acceptable range along with the possibility to adjust the Hemocue device should be reconsidered for future product developments of the Hemocue devices.

In contrast to our study, which found that venous Hb levels were slightly higher than capillary Hb concentrations using HemoCue Hb301, some previous studies have reported that capillary Hb by HemoCue was higher than venous Hb concentrations by HemoCue.^26 36^ The difference in Hb concentrations between capillary and venous blood samples has been explained by the fact that a capillary blood drop reflects the content of blood from various loop capillaries and small arterioles and venules, and a venous sample reflects the blood coursing through the veins, heart and arteries.^26^ A high within-subject variability (biological variability) of capillary blood samples compared with venous blood has previously been documented.^37^ However, our study does not fully support this hypothesis because we also found that venous Hb levels determined by HemoCue Hb301 were higher than venous Hb concentrations determined by an automated haematology analyser, implying that the primary difference found in our study was due to the analytical method used. Nevertheless, we found a small but significant difference between capillary and venous Hb concentrations by HemoCue Hb301, which may be due to improper capillary sampling such as excessive finger squeezing or partial filling of the microcuvette.^17^

Our finding that capillary Hb concentrations by HemoCue Hb301 are significantly higher than venous Hb concentrations obtained with two different automated haematology analysers is consistent with some previous studies in pregnant women in Sudan^19^ and Brazil,^36^ blood donors in the USA^26^ and different population subgroups (preschool children, school children, pregnant women, non-pregnant women and men) in Ghana,^25^ but not with studies in women with genetic Hb disorders in Cambodia,^21^ adults and children in Mexico,^29^ blood donors in Ireland^38^ and children in South Africa.^30^ Time and position during blood collection^39 40^ have been suggested to explain discrepancies between different devices and to influence the variability in Hb levels obtained by HemoCue. An additional source of error may be inadequate mixing of venous blood in the blood collection tube, which may lead to a reading of relatively more plasma rather than red blood cells by the automated analyser and would result in a lower Hb concentration. However, this may be less likely in our study since we found consistent results with automated haematology analysers run by different technicians based in two independent laboratories. To minimise technical errors we emphasised the correct blood collection and processing for each analytical method during training and continued supervision.

Direct comparison of our findings with previous studies is complicated by several factors. First, we used the HemoCue Hb301, while other studies used either the HemoCue B-Hemoglobin or HemoCue Hb201, which employs different biochemical methods to determine Hb concentrations. In addition, we used Sysmex XT-1800i and Mindray BC-3000Plus automated analysers, whereas others used other instruments. Other factors that differ across studies are the study settings, the participants’ age range and ethnicity and whether Hb was assessed on fasting or non-fasting blood.^41^ Our study included very young children (age ranged from 6 to 32 months old), while other studies included older children (1–4 years old^25^ or 6–8 years old^30^) and found HemoCue to be comparable with the Sysmex KX21N and the Siemens Advia 2120 analysers, respectively. Indeed, our results suggest that the age of the study population and the anaemia prevalence are potentially important factors. Specifically, we found that the bias (difference between mean Hb concentrations using the two methods) is similar in 12-month to 17-month and 18-month to 24-month children, but significantly lower than that of children 6–11 months of age; potential explanations of this finding may be that the anaemia prevalence was higher in younger children or that blood collection may be more challenging among very young children. Another factor complicating the comparison with other studies is represented by the range of Hb concentrations in the study population. In a study of over 36 000 paired capillary and venous samples of adult blood donors, venous Hb levels by automated haematology analyser were consistently higher than capillary levels by HemoCue when the Hb concentrations were in the lower part of the normal non-anaemic range.^38^ Similarly, a study in Cambodian women with genetic Hb disorders reported that the HemoCue (HemoCue Hb 201+Hemocue AB) appears to underestimate Hb concentrations in capillary blood as compared with venous Hb by Sysmex at lower Hb concentrations and to overestimate Hb concentrations at higher Hb concentrations, resulting in false positives in the diagnosis of anaemia.^21^ This is in contrast to our study, which found that HemoCue Hb301 overestimated capillary Hb levels at lower Hb concentrations, resulting in a lower prevalence of anaemia.

As mentioned above, the two HemoCue Hb301 devices used in the field during enrolment and endline assessments provided comparable results, while a third back-up device yielded significantly different concentrations. Similarly, inconsistent measurements were found using 12 different HemoCue Hb301 devices in a methodological study of women in Cambodia.^42^ Further investigation is needed to determine inherent variability among different HemoCue Hb301 devices, and results should be routinely compared when multiples devices are deployed in a single study.

One strength of this study is its large sample size, which allowed us to explore the agreement between methods by age group. In addition, we ran different sets of analyses to compare capillary and venous Hb using HemoCue Hb301, and we included regular quality control and a methodological comparison of three individual HemoCue Hb301 devices owned by the project. Moreover, the capillary and venous blood draws occurred within a few minutes of each other for each child. This study also has some limitations. It has been suggested that the HemoCue device may be more accurate in Hb concentration determination than automated analysers^43^ given that the latter requires a sample dilution, while HemoCue assesses Hb directly and is not affected by changes in turbidity. We were not able to confirm this statement as we did not assess Hb concentrations using the cyanmethemoglobin method, which is considered the reference procedure by the International Committee for Standardization in Hematology and WHO.^10^ We were also not able to compare the accuracy of the two automated haematology analysers against each other in the same sample of children because the respective subsamples were collected for different aspects of the Lao Zinc Study. It is worth mentioning that a WHO consultation on Hb assessment is ongoing and will likely shed new light on guidelines and recommendations for Hb assessment and interpretation.

## Conclusion

To date, our study is the largest study comparing capillary Hb concentration assessed by HemoCue Hb301 with venous Hb concentrations determined by automated analysers in young children. Regardless of the type of blood sample, the HemoCue device showed poor agreement compared with two automated haematology analysers, resulting in significantly different anaemia prevalences. The HemoCue is useful in many different settings and remains a widely used method in field settings as it has several advantages and is relatively inexpensive compared with automated haematology analysers. However, given the difference in anaemia prevalences found with the different methods, further research is needed to better understand potential sources of error in the Hb assessment by HemoCue with the aim to better train phlebotomists and implement appropriate standardised procedures.

**Take home messages**

- The Hemocue Hb301 device showed poor agreement compared with two automated haematology analysers.
- Capillary haemoglobin (Hb) concentration by Hemocue Hb301 was higher than venous Hb concentration by automated haematology analyser.
- Venous Hb concentration by Hemocue Hb301 was higher than capillary Hb concentration by Hemocue Hb301.
- Methodological differences resulted in a significantly lower anaemia prevalence by Hemocue.
