# Supplementary material for: Comparison of haemoglobin assessments by HemoCue and two automated haematology analysers in young Laotian children
Source: J Clin Pathol. 2017 Dec 2;71(6):532–8. doi: 10.1136/jclinpath-2017-204786 (PMC5969348; doi:10.1136/jclinpath-2017-204786)
Supplement: Supplementary data [file jclinpath-2017-204786supp001.docx]

**Table S1:** Hemoglobin concentration and anemia prevalence using HemoCue® and automated hematology analyzers individually

|  | **Hospital-based automated hematology analyzer** | | | **Field-based automated hematology analyzer** | | |
| --- | --- | --- | --- | --- | --- | --- |
|  | **HemoCue® Hb301** | **XT-1800, Sysmex** | **p-value** | **HemoCue® Hb301** | **BC-3000Plus, Mindray Med. Internat. Ltd** | **p-value** |
| **Participants*, n (%)** | 633 (42.6) | |  | 854 (57.4) | |  |
| **Hb (mean ± SD), g/L** | 110.7 ± 9.7 | 107.4 ± 12.5 | <0.0001 | 106.7 ± 10.4 | 98.4 ± 12.2 | <0.0001 |
| **Anemia prevalence, %** | 46.0 | 59.4 | <0.0001 | 59.5 | 84.7 | <0.0001 |
| **Bias (95% limits of agreement)** | 3.2 (-14.1, 20.5) | |  | 8.3 (-8.4, 24.9) | |  |
| **r (Correlation coefficient)** | 0.71 | | <0.0001 | 0.73 | | <0.0001 |
| **Sensitivity, %** | 69.4 | |  | 66.8 | |  |
| **Specificity, %** | 88.3 | |  | 80.9 | |  |

*Each comparison is done in a different sub-group of children


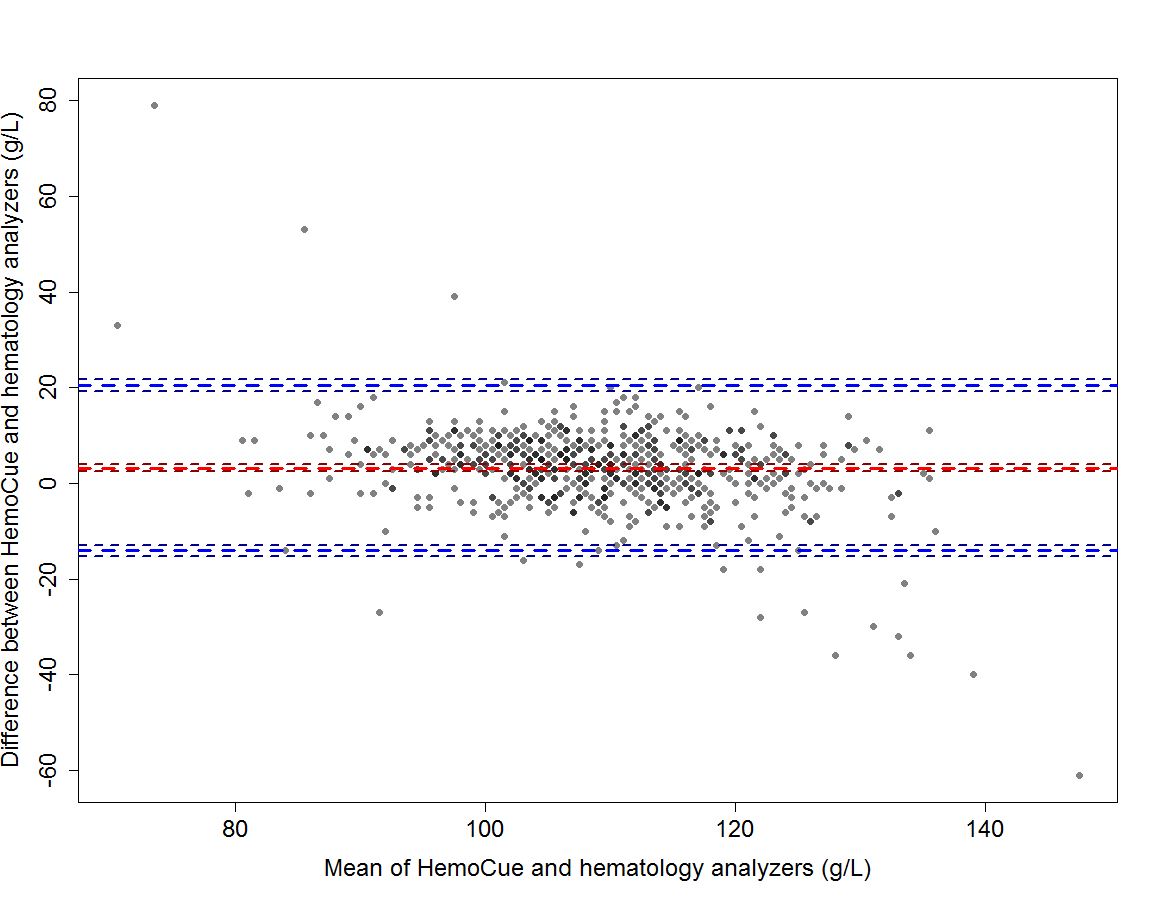


**Figure S1**: Bland-Altman plot showing agreement in hemoglobin concentration assessed by HemoCue®Hb301 and by the automated hematology analyzer XT-1800 by Sysmex


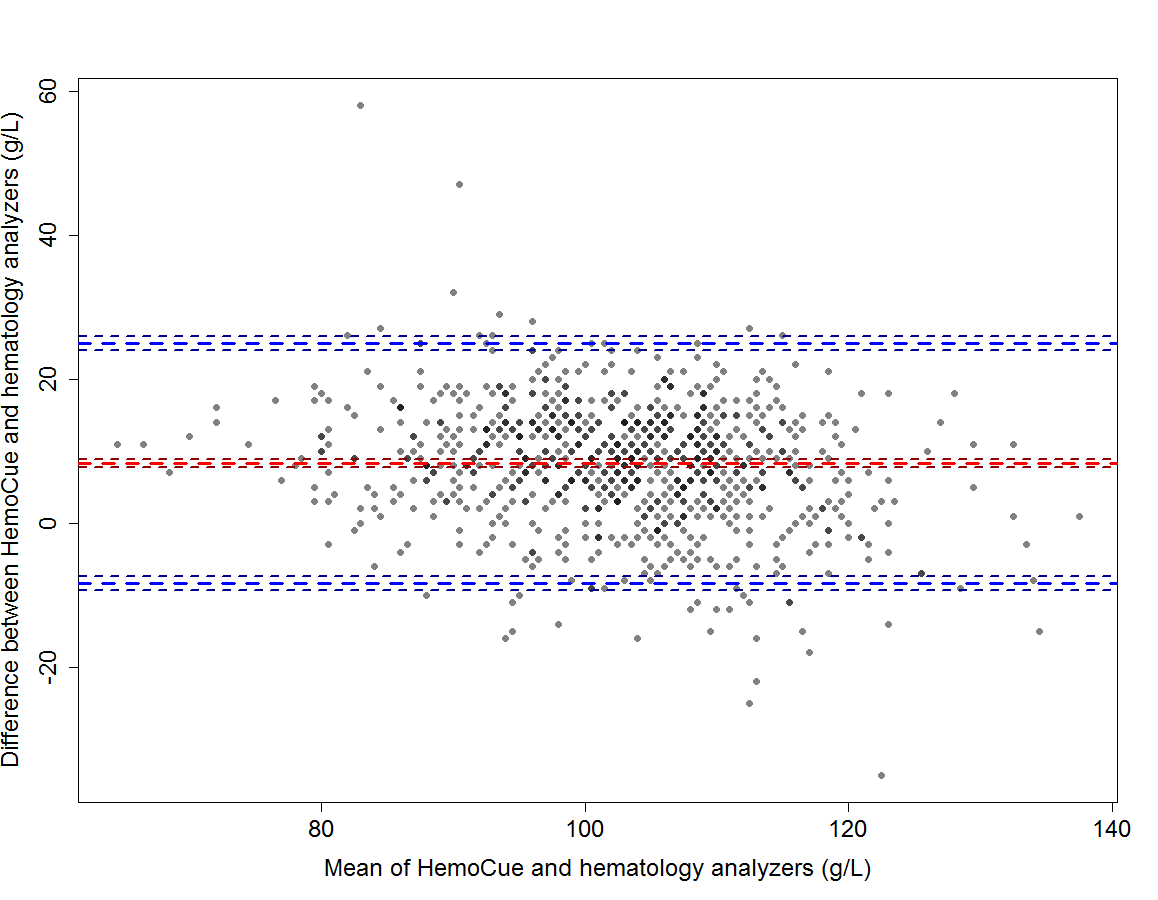


**Figure S2**: Bland-Altman plot showing agreement in hemoglobin concentration assess by HemoCue®Hb301 and BC-3000Plus, Mindray automated hematology analyzer

**Table S2**: Hemoglobin concentration and anemia prevalence by age groups in young Laotian children using two different methods of assessment

| Age, months | N | Hemoglobin (mean ± SD), g/L | | | | Anemia prevalence n (%) | | |
| --- | --- | --- | --- | --- | --- | --- | --- | --- |
|  |  | HemoCue® Hb301 | Automated analyzers* | p-value | Bias (95% CI), g/L | HemoCue® Hb301 | Automated analyzers* | p-value |
| 6-11 | 523 | 106.2 ± 10.2 | 98.9 ± 12.2 | <0.0001 | 7.3 (6.6-8.0) | 318 (60.8) | 436 (83.4) | <0.0001 |
| 12-17 | 502 | 107.8 ± 10.0 | 101.9 ± 13.6 | <0.0001 | 5.9 (5.1-6.7) | 286 (57.0) | 286 (73.5) | <0.0001 |
| 18-24 | 462 | 111.4 ± 9.9 | 106.5 ± 12.4 | <0.0001 | 5.0 (4.1-5.8) | 195 (42.2) | 195 (63.6) | <0.0001 |

* XT-1800i by Sysmex and BC-3000Plus by Mindray Medical International Ltd


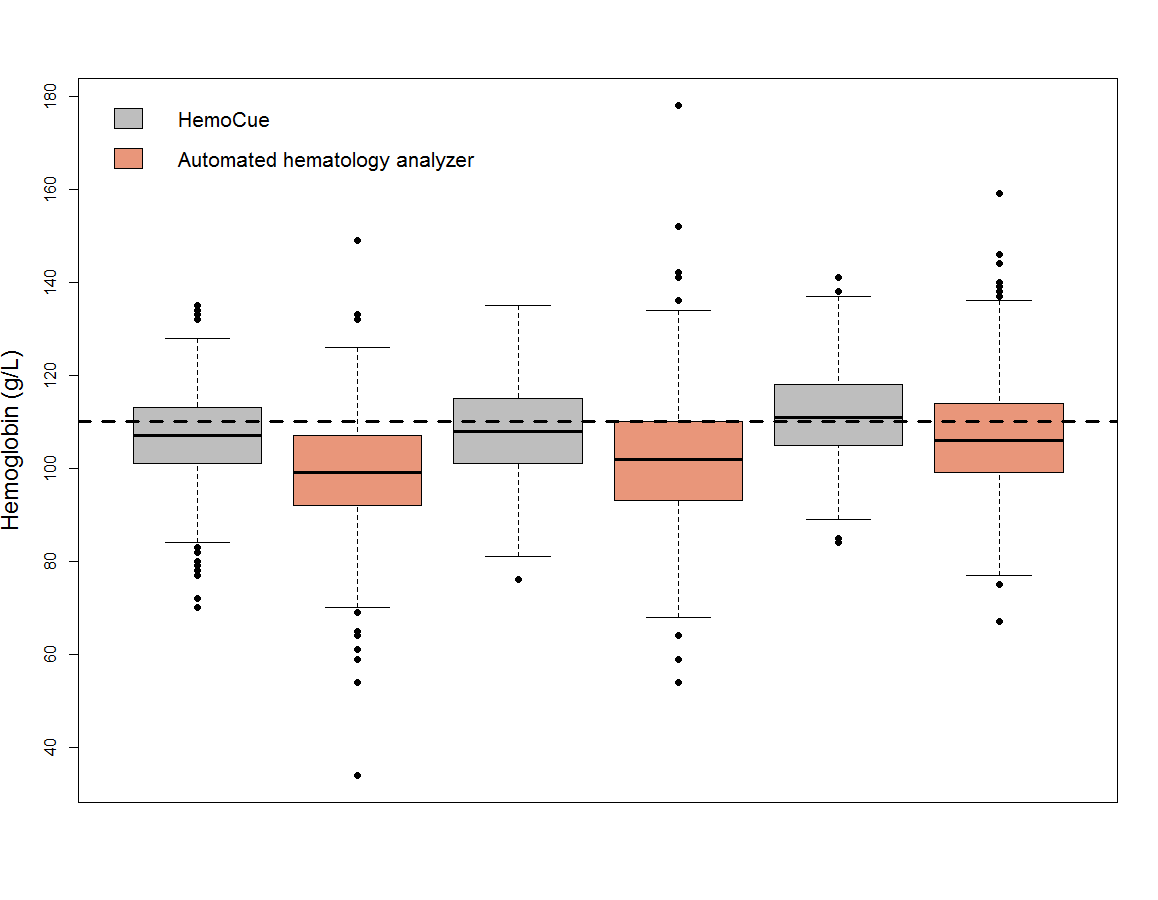


18-24 mo

12-17 mo

06-11 mo

**Figure S3**: Box plots comparing the distribution of hemoglobin levels by age group using HemoCue®Hb301 and the automated analyzers combined (XT-1800i by Sysmex and BC-3000Plus by Mindray Medical International Ltd).

*Dash line represents the Hb cut-off value for anemia in children of 6-59 months of age (Hb < 110g/L)

**Table S3:** Hb measurements provided by the three different HemoCue® Hb301 devices

|  | **HemoCue® #1 Vs HemoCue® #2** | **HemoCue® #1 Vs HemoCue® #3** | **HemoCue® #2 Vs HemoCue® #3** |
| --- | --- | --- | --- |
| **Hb (mean ± SD), g/L** | 112.9 ± 8.8 vs 113.2 ± 6.9 | 112.9 ± 8.8 vs 117.1 ± 9.4 | 113.2 ± 6.9 vs 117.1 ± 9.4 |
| **Difference (95% CI), g/L** | -0.3 (-1.8, 1.3)* | -4.2 (-7.2, -1.2)** | -3.9 (-6.6, -1.3)** |
| **Anemia prevalence, %** | 41.4 vs 34.5* | 41.4 vs 20.7* | 34.5 vs 20.7* |
| **r (Correlation coefficient)** | 0.89*** | 0.62*** | 0.67*** |
| **Bias (95% limits of agreement)** | -0.28 (-8.23, 7.68) | -4.20 (-19.78, 11.36) | -3.93 (-17.71, 9.85) |

*Non statistically significant

**p < 0.05

***p < 0.001
